# Pharmacoeconomic analysis of antifungal therapy for primary treatment of invasive candidiasis caused by *Candida albicans* and non-*albicans Candida* species

**DOI:** 10.1186/s12879-017-2573-8

**Published:** 2017-07-10

**Authors:** Huang-Tz Ou, Tsung-Ying Lee, Yee-Chun Chen, Claudie Charbonneau

**Affiliations:** 1 Institute of Clinical Pharmacy and Pharmaceutical Sciences, College of Medicine, National Cheng Kung University, Tainan, Taiwan; 2 Department of Pharmacy, College of Medicine, National Cheng Kung University, Tainan, Taiwan; 3 Department of Pharmacy, National Cheng Kung University Hospital, Tainan, Taiwan; 4 Department of Internal Medicine, National Taiwan University Hospital, Taipei, Taiwan; 5 Department of Medicine, College of Medicine, National Taiwan University, Taipei, Taiwan; 6 Pfizer International Operations, Paris, France

**Keywords:** Pharmacoeconomics, Antifungal therapy, Candidemia, Anidulafungin, Echinocandin, Fluconazole

## Abstract

**Background:**

Cost-effectiveness studies of echinocandins for the treatment of invasive candidiasis, including candidemia, are rare in Asia. No study has determined whether echinocandins are cost-effective for both *Candida albicans* and non-*albicans Candida* species. There have been no economic evaluations that compare non-echinocandins with the three available echinocandins. This study was aimed to assess the cost-effectiveness of individual echinocandins, namely caspofungin, micafungin, and anidulafungin, versus non-echinocandins for *C. albicans* and non-*albicans Candida* species, respectively.

**Methods:**

A decision tree model was constructed to assess the cost-effectiveness of echinocandins and non-echinocandins for invasive candidiasis. The probability of treatment success, mortality rate, and adverse drug events were extracted from published clinical trials. The cost variables (i.e., drug acquisition) were based on Taiwan’s healthcare system from the perspective of a medical payer. One-way sensitivity analyses and probability sensitivity analyses were conducted.

**Results:**

For treating invasive candidiasis (all species), as compared to fluconazole, micafungin and caspofungin are dominated (less effective, more expensive), whereas anidulafungin is cost-effective (more effective, more expensive), costing US$3666.09 for each life-year gained, which was below the implicit threshold of the incremental cost-effectiveness ratio in Taiwan. For *C. albicans*, echinocandins are cost-saving as compared to non-echinocandins. For non-*albicans Candida* species, echinocandins are cost-effective as compared to non-echinocandins, costing US$652 for each life-year gained. The results were robust over a wide range of sensitivity analyses and were most sensitive to the clinical efficacy of antifungal treatment.

**Conclusions:**

Echinocandins, especially anidulafungin, appear to be cost-effective for invasive candidiasis caused by *C. albicans* and non-*albicans Candida* species in Taiwan.

**Electronic supplementary material:**

The online version of this article (doi:10.1186/s12879-017-2573-8) contains supplementary material, which is available to authorized users.

## Background

Invasive candidiasis (IC), including candidemia, is associated with considerable morbidity and mortality. Managing IC is costly, with an additional healthcare expenditure of nearly US$300 million annually [1]. Our previous study showed that healthcare-associated infection due to *Candida albicans* was associated with a mean additional hospital stay of 18.4 ± 28.5 days and an extra cost of up to US$6584 ± 11,467 when amphotericin B deoxycholate (d-AmB) and fluconazole were the only two parenteral antifungal agents [2]. Current international guidelines [3–6] suggest the use of echinocandins (caspofungin, micafungin, and anidulafungin) for the primary treatment of IC because of their cidal activity, rarity of resistance, safety profile, and better clinical outcomes compared with those of fluconazole and d-AmB [7, 8].

However, echinocandins have higher drug acquisition and administration costs. Studies from Spain, the United Kingdom, and Australia have shown that treatment with anidulafungin is cost-effective as compared to that with fluconazole [9–11]. However, cost-effectiveness studies of echinocandins for treating IC are rare in Asia. Also, published economic evaluations [9, 10] compare fluconazole with anidulafungin only. In general, echinocandins are similar with respect to their broad spectrum of activity and in vitro activity against *C. albicans* and non-*albicans Candida* spp., but each has its own unique features and drug acquisition cost. There is a lack of a thorough analysis comparing the economic advantages and disadvantages of the three available echinocandins.

A change in the epidemiology of IC has been witnessed in recent decades, with a progressive shift from a predominance of *C. albicans* toward a predominance of non-*albicans Candida* spp. (including *C. glabrata* and *C. krusei*, which are less susceptible or resistant to fluconazole) [12]. In addition, the distribution of *Candida* species varies by geographic and healthcare factors [13]. However, no study has determined whether echinocandins are cost-effective for both *C. albicans* and non-*albicans Candida* spp. as compared to fluconazole.

In this study, we assess the cost-effectiveness of individual echinocandins versus fluconazole in terms of either reduced hospital stay or better clinical outcomes. Subgroup analyses were conducted for *C. albicans* and non-*albicans Candida* spp., respectively.

## Methods

This was a pharmacoeconomic study, which utilized the secondary data reported from published studies, so ethics approval was waived.

### Perspective

This study was carried out based on Taiwan’s National Health Insurance from a single-payer perspective, and included only direct medical costs (drug acquisition, hospitalization costs, and treatment of major adverse effects such as renal toxicity).

### Model specifications and assumptions

**Fig. 1:**
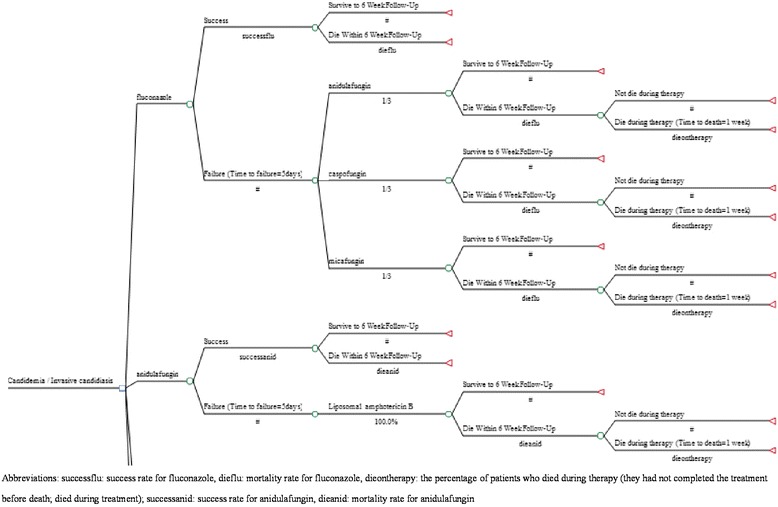
Decision model: fluconazole vs. echinocandins (i.e., anidulafungin) for treating candidemia/invasive candidiasis

The applied decision-analytic tree was based on the anidulafungin cost-effective model [11], which represents the treatment pathway for patients receiving different types of antifungal treatment (Fig. 1). This model was described by Auzinger et al. in detail [11]. The anidulafungin cost-effectiveness model [11] was developed from the perspective of the United Kingdom to examine the costs and outcomes of antifungal treatment for IC based on the European Society for Clinical Microbiology and Infectious Diseases guidelines, which are consistent with the clinical practice for managing IC in Taiwan. Based on Reboli et al.’s study [8], the anidulafungin cost-effective model [11] assumes that the average weight for patients receiving liposomal amphotericin B is 76.4 kg ± 25.5 kg. Based on drug labeling in Taiwan, the loading and maintenance doses of micafungin were both set to 100 mg for candidemia [14]. The dosing forms for the treatments of interest in this study were assumed: fluconazole: 400 mg once daily, micafungin: 100 mg once daily, caspofungin: loading dose: 70 mg and maintenance dose: 50 mg once daily, anidulafungin: loading dose: 200 mg and maintenance dose: 100 mg once daily.

Model 1 was adapted from the anidulafungin model [11], showing a person with fluconazole or echinocandins (i.e., caspofunglin, micafunigin, anidulafungin). If the treatment was successful, then the person continued the intravenous (IV) antifungal treatment for 14 days. If fluconazole had failed, the person was switched to one of the echinocandins (one-third of patients were treated with anidulafungin, caspofungin, and micafungin, respectively [11]). For those treated with anidulafungin initially, liposomal amphotericin B was the rescue agent after failure. Those who had experienced clinical failure and were switched to another type of treatment, which is assumed to clear the infection immediately, received an additional 14 days of second-line treatment and were followed for 6 weeks or until death. Patients who died within 6 weeks of treatment were classified as either “did not die during therapy” (they had completed the treatment but died) or “died during therapy” (they had not completed the treatment before death; died during treatment).

Model 2, whose structure was similar to that of model 1, was designed to capture the downstream economic consequences of using echinocandins or non-echinocandins as primary therapy for *C. albicans* or non-*albicans Candida* spp. If the treatment had failed, liposomal amphotericin B was used as the alternative. The users of liposomal amphotericin B were observed for 6 weeks or until death (Additional file 1: Figure S1).

### Model parameters

Table 1 shows the parameters of treatment efficacy (i.e., success rate, mortality), percentage of patients that die during therapy, life expectancy, length of IV treatment for patients with treatment success and then survival, and drug adverse events (i.e., nephrotoxicity), which were primarily derived from the literature, costs (i.e., drug acquisition costs, length of hospital stay (LOS) in intensive care unit, and other types of hospital stay), which were based on Taiwan’s healthcare system [15], and parameters related to LOS, which were based on expert opinions on clinical practice in Taiwan [16, 17]. A four-member expert panel comprising clinicians and researchers within Taiwan’s healthcare system with significant experience in infectious diseases provided consensus opinions for data (i.e., LOS) not available from the literature.

**Table 1:** Summary of model parameters

Variable	Value [ref.]
Probability of success rate (%)	
*All species*	
Fluconazole	63 [22]
Anidulafungin	77.49 [22]
Micafungin	75.98 [22]
Caspofungin	76.10 [22]
*C. albicans specific*	
Echinocandins	81 [8]
Non-echinocandins	22^a^ [7]
Non-*albicans specific*	
Echinocandins	71 [8]
Non-echinocandins	71^a^ [7]
Probability of mortality (%)	
All species	
Fluconazole	28.44 [22]
Anidulafungin	20.75 [22]
Micafungin	39.16 [22]
Caspofungin	33.83 [22]
*C. albicans* specific	
Echinocandins	19 [Pfizer, Data in file]
Non-echinocandins	35^a^ [7]
Non-*albicans* specific	
Echinocandins	26 [Pfizer, Data in file]
Non-echinocandins	50^a^ [7]
Percentage that die during therapy (%)	23.26 [25]
Life expectancy, mean (years)	9.12 [9]
Length of IV treatment for patients with treatment success and then survival (days)	14 [6, 18]
Major drug adverse events	
Nephrotoxicity probability for amphotericin B (%)	33.7 [26]
Relative risk of nephrotoxicity of fluconazole compared with that of amphotericin B	0.22 (95% CI, 0.15–0.32) [22]
Relative risk of nephrotoxicity of echinocandins compared with that of amphotericin B	0.31 (95% CI, 0.17–0.57) [22]
Additional LOS for nephrotoxicity, mean (days)	7 (95% CI, 5.7–8.4) [27]
Time required to determine clinical failure (days)	5 [Expert opinion]
Follow up period (weeks)	6 [Expert opinion]
Time to death for patients who had treatment success but died before 6-week follow-up, mean (weeks)	3.25 [8]
Time to death for patients who had treatment failure that died before 6-week follow-up, mean (weeks)	3.25 [8]
Average time to death during therapy (weeks)	1 [Expert opinion]
ICU LOS (days)^b^	
Success and then survival	7 [Expert opinion]
Success and then death	7 [Expert opinion]
Failure and then survival	14 [Expert opinion]
Failure and then death	14 [Expert opinion]
Other hospital LOS (days)^b^	
Success and then survival	23 [Expert opinion]
Success and then death	23 [Expert opinion]
Failure and death survival	23 [Expert opinion]
Failure and then death	23 [Expert opinion]
Cost (US$)	
Loading dose cost	
Fluconazole 400 mg/day	43.76 [28]
Anidulafungin 200 mg/day	164.48 [28]
Micafungin 100 mg/day	108.85 [28]
Caspofungin 70 mg/day	517.70 [28]
Liposomal amphotericin B 3 mg/kg for a patient with 76.4 kg ± 25.5 kg	962.61 [28]
Echinocandins	263.67^c^ [28]
Non-echinocandins	43.76^d^ [28]
Maintenance dose cost	
Fluconazole 400 mg/day	43.76 [28]
Anidulafungin 100 mg/day	82.24 [28]
Micafungin 100 mg/day	108.85 [28]
Caspofungin 50 mg/day	258.85 [28]
Liposomal amphotericin B 3 mg/kg for a patient with 76.4 kg (± 25.5 kg)	962.61 [28]
Echinocandins	149.97^c^ [28]
Non-echinocandins	43.76^d^ [28]
ICU cost per day	203.39 [28]
Other hospital cost per day	43.27 [28]

*Abbreviations: LOS* length of hospital stay, *ICU* intensive care unit

^a^Transformation of data from literature [7, 8]. Of note, because there appears to be no significant difference in the treatment success rate among the three echinocandins, we used the data (i.e., treatment success) for anidulafungin for *C. albicans* and non-*albicans,* respectively, in Reboli et al.’s study [8] for “echinocandins” for *C. albicans* and non-*albicans*. For example, Reboli et al.’s study [8] reported a success rate of anidulafungin treatment for *C. albicans* of 0.81 and Adnes et al.’s study [7] showed that the odds ratio for echinocandin treatment success as compared to that for non-echinocandins (including polyenes [amphotericin B and liposomal amphotericin B] and triazoles [fluconazole and voriconazole]) for *C. albicans* is 3.7, so the success rate of non-echinocandins was estimated to be 0.22. Similarly, Reboli et al.’s study [8] reported a success rate of anidulafungin treatment for non-*albicans* (including *C. glabrate, C. parapsilosis, C. tropicalis*, and other species) of 0.71 and Adnes et al.’s study [7] showed that the odds ratio for echinocandins treatment success as compared to that of non-echinocandins for non-*albicans* is 1, so the success rate of non-echinocandins for non-*albicans* was estimated to be 0.71. This transformation was also applied to estimate mortality rates for echinocandins and non-echinocandins for *C. albicans* and non-*albicans,* respectively

^b^According to clinical practice in Taiwan [16, 17], experts assumed an average of 30 days for total length of hospital stay (LOS), of which 7 days are for stay in intensive care unit (ICU) and 23 days are for other hospital stay

^c^Average drug cost of echinocandins, including anidulafungin, micafungin, and caspofungin

^d^The cost of non-echinocandins refers to drug cost of fluconazole

### Cost-effectiveness analysis

The incremental cost-effectiveness ratio (ICER) was calculated as the ratio of the difference in medical and drug acquisition costs to the difference in life-years (LY) gained and is expressed in US dollars per LY gained (US$/LY). Noticeably, the LY gained is the difference or incremental value in the LY between two treatment groups. The LY for each treatment group was an expected value aggregated from two components in the decision model (i.e., Fig. 1) (1) time to death in the mortality cases within 6-weeks of follow-up, and (2) life expectancy for the survived cases during 6-weeks of follow-up. First, the time to death (presented in LY) in the mortality cases was obtained directly from Reboli et al.’s study [8]. The life expectancy for the survived cases was estimated in the following steps: (a) assume an average age of patients with IC were approximately 58 years old [8], (b) assume the remaining life expectancy of a 58-year-old person (without IC) is 25.29 years old in the United Kingdom (from Office of National Statistics), (c) the life expectancy was then adjusted by using the reported relative risk of death of 0.51 for sepsis survivors [18], (d) based on the known life table of general population (without IC) and the relative risk of death for sepsis, the expected life expectancy for a sepsis survivor was estimated as 12.9 years. This value was further discounted at an annual rate of 5% for 40 years of follow-up period, which turn out to 9.12 years as the remaining life years for the survived cases within 6 weeks of follow-up.

There is no defined willingness-to-pay threshold for health interventions in Taiwan. Therefore, according to the World Health Organization’s recommendations [19], the treatment was considered as cost-effective if the cost of one LY gained was less than three times the per capita national gross domestic product (GDP). Taiwan’s per capita GDP was US$22,355 in 2015 [20], so the implicit cost-effectiveness threshold was calculated to be US$67,065 per LY gained. All costs are expressed in 2015 US dollars.

### Sensitivity analysis

A one-way sensitivity analysis was carried out for efficacy and cost data in the models to determine the impact of uncertainty on model outcomes. A probabilistic sensitivity analysis based on 10,000 Monte Carlo simulations was also performed to assess the simultaneous effect of uncertainty on model results. The gamma, beta, and triangular distributions were used for the price, costs, transition probabilities, and other parameters, while the outcome variables were assumed to be normally distributed [21]. A cost-effectiveness acceptability curve was plotted using the probability of the treatment being cost-effective at a threshold value of willingness-to-pay per LY gained in Taiwan. TreeAge Pro 2016, R1.2 (TreeAge Software, Inc., MA, USA) was used for these economic analyses.

## Results

### Base case analysis

For treating IC, including candidemia, due to any *Candida* species, our analysis estimated that as compared to fluconazole, micafungin and caspofungin are less effective but more expensive (dominated), whereas anidulafungin is more effective and more expensive (cost-effective), costing US$3666.09 for each LY gained under the assumption that the length of IV treatment for success and survival is 14 days (Table 2). Anidulafungin remains cost-effective, costing US$8015.39 for each LY gained, under the assumption that the length of IV treatment for success and survival is 30 days (Additional file 1: Table S1). Furthermore, as compared to anidulafungin, micafungin and caspofungin both are dominated (Additional file 1: Table S2).

**Table 2:** Incremental cost-effectiveness ratio (ICER) (assuming length of IV treatment for success and survival is 14 days)

Candidemia/invasive candidiasis	First-line treatment	Total cost	Incremental cost	Total life-years	Incremental life-years	ICER
All species	Fluconazole (ref.)	4229	-	6.52	-	-
	Anidulafungin	6799	2570	7.23	0.70	3666
	Micafungin	7171	2942	5.55	−0.98	−3011
	Caspofungin	9211	4983	6.03	−0.49	−10,140
*Candida albicans*	Non-echinocandins (ref.)	13,972	-	5.97	-	-
	Echinocandins	7175	−6797	7.39	1.42	−4796
Non-*albicans*	Non-echinocandins (ref.)	7043	-	4.56	-	-
	Echinocandins	8469	1426	6.75	2.19	652

Cost value is presented in 2015 US dollars

Table 2 also indicates that for *C. albicans*-infected patients, echinocandins are more effective and less expensive as compared to non-echinocandins, implying that the former are likely to be cost-saving. For non-*albicans Candida* spp., echinocandins are more effective but more expensive (cost-effective) as compared to non-echinocandins, costing US$652 for each LY gained.

We also utilized treatment efficacy data at different time points from Reboli et al.’s study [8] to examine the robustness of our results. We found that anidulafungin is more effective and more expensive as compared to fluconazole, costing US$6310.01 for each LY gained (when the input is the success rate at 6 weeks follow-up) and US$3492.01 for each LY gained (when input is the success rate at the end of IV treatment) (Additional file 1: Table S3).

### Sensitivity analyses

**Fig. 2:**
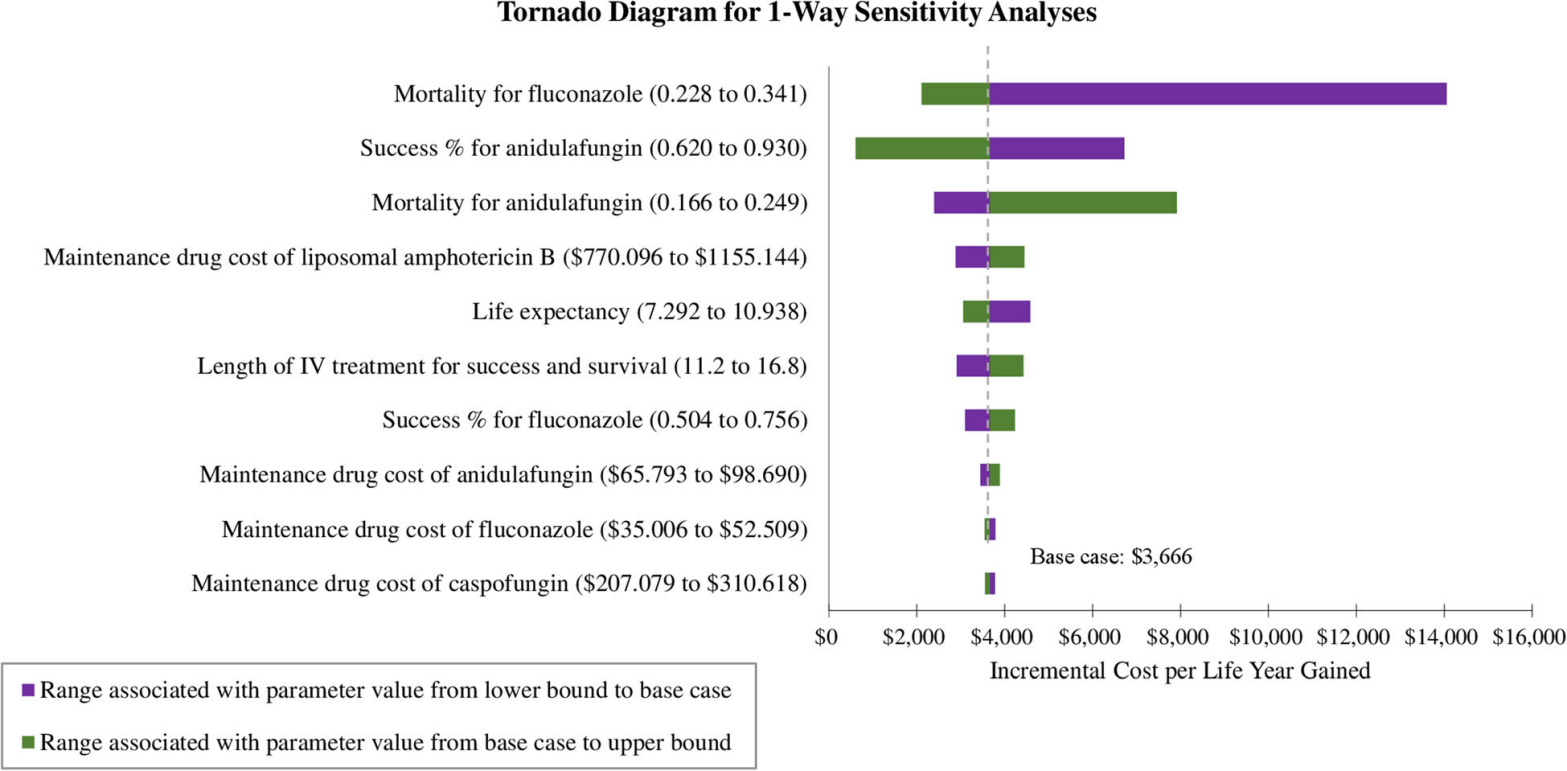
Tornado sensitivity analysis of anidulafungin vs. fluconazole for Candidemia/invasive candidiasis (x axis presents values of incremental cost-effectiveness ratio; ICER, incremental cost per life-year gained. Cost value is presented in 2015 US dollar)

A tornado diagram showed that the ICER value is most sensitive to “mortality rate for fluconazole” (Fig. 2). In the cost-effectiveness analysis, the most influential variable for *C. albicans*-infected patients is “mortality rate for non-echinocandins” and that for non-*albicans Candida*-infected patients is “success rate for non-echinocandins” (Additional file 1: Figure S2). Of note, when the success rate for non-echinocandins for non-*albicans Candida* infection is less than 0.606, using echinocandins is cost-saving. Therefore, the ICER results are sensitive to the efficacy parameters of treatment (i.e., success rate or mortality rate associated with treatment).

**Fig. 3:**
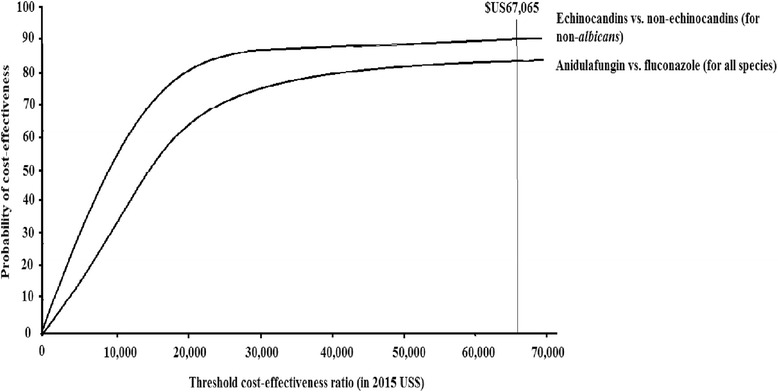
Cost-effectiveness acceptability curve

The cost-effectiveness acceptability curve showed that anidulafungin, as compared to fluconazole, has an 82% probability of being cost-effective at a threshold of three times the per capita GDP of Taiwan (US$67,065; Fig. 3). Echinocandins for non-*albicans Candida*-infected patients has an 89% probability of being cost-effective at a threshold of three times the per capita GDP of Taiwan, as compared to non-echinocandins.

Because efficacy data of non-echinocandins were converted based on Andes et al.’s study [7], we further conducted one-way sensitivity analysis for the treatment success rate for non-echinocandins for *C. albicans* infection to ensure the robustness of our results (Additional file 1: Figure S3). We found that the direction of cost-effectiveness results changed when different treatment success values for non-echinocandins were assumed. Specifically, when the treatment success rate for non-echinocandins was assumed to be 0.22, the ICER was estimated to be -US$4796 (our base case analysis), implying cost saving when using echinocandins instead of non-echinocandins. When the treatment success rate for non-echinocandins was assumed to be the same as that for echinocandins (0.81), the ICER value was US$1029. Since $1029 is below Taiwan’s cost-effectiveness threshold, using echinocandins under this assumption is still considered to be cost-effective and acceptable. When the treatment success rate for non-echinocandins is 0.706, the ICER value is US$0, indicating no difference between echinocandins and non-echinocandins; i.e., the costs of these two treatments are the same. Even if the average cost of individual echinocandins was used as the drug acquisition cost for echinocandins, our sensitivity analyses showed that cost-effectiveness results were not sensitive to drug acquisition cost for echinocandins. Therefore, the direction of ICER is likely to stay the same regardless of drug acquisition cost of individual echinocandins.

## Discussion

To the best of our knowledge, this is the first study to comprehensively assess the cost-effectiveness of echinocandins versus non-echinocandins such as fluconazole for different species (*C. albicans* vs. non- *albicans Candida* spp*.*) of IC in Taiwan. Our results indicate that among echinocandins, only anidulafungin is cost-effective as compared to fluconazole. For *C. albicans*-infected patients, the use of echinocandins is likely to be cost-saving as compared to the use of non-echinocandins. For non-*albicans Candida*-infected patients, there is an 82% chance of the outcome favoring echinocandins.

Three cost-effectiveness studies from other countries compared anidulafungin with fluconazole for IC, providing findings that are consistent with our study. Neoh et al.’s study based on an Australian hospital perspective and Reboli et al.’s trial data [8] indicated that, as compared to fluconazole, anidulafungin was associated with an ICER of AU$25,740 per LY gained, which was under the Australian ICER threshold, suggesting that anidulafungin is a cost-effective agent [9]. Our additional analyses, which applied Reboli et al.’s trial data [8], showed consistent results (Additional file 1: Table S3) with those in Neoh et al.’s study [9]. Grau et al.’s study from Spain showed that anidulafungin was cost-saving over fluconazole, with a higher clinical success (74% vs. 57%) at a lower total medical cost (€40,047 vs. €41,350) and that the clinical efficacy of antifungal treatment was the most influential factor in the cost-effectiveness analysis [10], which is consistent with the results of the sensitivity analyses in the present study (Fig. 2). Auzinger et al.’s study, from the perspective of the United Kingdom National Health Service and Personal and Social Services, showed that anidulafungin was cost-effective as compared to fluconazole (ICER: £813 per LY gained) and cost-saving versus caspofungin and micafungin [11]. However, none of these studies analyzed the cost-effectiveness of antifungal treatments for specific species (i.e., *C. albicans*).

Fluconazole has been commonly used for systemic *Candida* infections; however, selected *Candida* spp. are intrinsically resistant to or prone to develop resistance to fluconazole. In addition, fluconazole is inactive against *Candida* biofilm formation [6, 7]. Both may contribute to treatment failure. In contrast, echinocandins have very low resistance rates and are active against *Candida* biofilm. Echinocandins are associated with higher success rates as compared to those for fluconazole [7, 8]. The present study shows that, as compared to non-echinocandins, echinocandins are likely to be cost-effective for both *C. albicans* and non- *albicans Candida* species.

Among the three available echinocandins, anidulafungin is cost-saving as compared to caspofungin and micafungin because of its higher rate of survival combined with a higher probability of treatment success and lower total costs. Anidulafungin has shown better efficacy (i.e., treatment success) versus those of other echinocandins in a mixed treatment comparison [22]. Also, it does not require dose adjustments, which are required for caspofungin (according to hepatic function). Because anidulafungin is metabolized by slow chemical, rather than enzymatic, degradation, there is no need for dose titration in patients with renal or hepatic impairment. As compared to fluconazole, the use of anidulafungin costs US$8015 per LY gained and has an 89% probability of being cost-effective at a threshold of three times per capita GDP of Taiwan (US$67,065). Therefore, anidulafungin is a treatment option that allows better control of antifungal budgets and leads to better healthcare outcomes (i.e., LY gained) at lower total costs.

The advantage of the present study is that it takes into account the downstream economic consequences of failed first-line antifungal treatment and considerable adverse drug effects (i.e., nephrotoxicity). The findings of this study might be extrapolated to other countries with similar healthcare systems (i.e., universal healthcare insurance coverage). In addition, the efficacy data were based on randomized controlled trials [7, 8, 22]. The various sensitivity analyses indicate fair robustness of the conclusions of this study. The overall conclusion remained the same in an additional analysis that changed the assumption of the length of IV treatment for patients with treatment success and then survival (i.e., 14 days or 30 days). Also, subgroup analyses for *C. albicans* and non-*albicans Candida* spp. show consistent favoring cost-effectiveness results for outcome of echinocandins.

However, some potential limitations of this study need to be addressed. First, our decision-analytic tree that was based on the anidulafungin cost-effective model [11] might only present a simplified model of daily clinical practice. For example, our model and sensitivity analysis did not take into consideration the heterogeneity of the patient population. For example, current guidelines suggest echinocandins for moderately to severely ill patients (from intensive care unit vs. general ward) and neutropenic patients (vs. non-neutropenic) as pooled individual data showed better effectiveness compared to non-echinocandins. Also, since our efficacy data were based on Reboli et al.’s trial [8] that included predominantly non-neutropenic patients with IC, our economic results may not be generalizable to the population of neutropenic patients with IC. Thus, the current data might underestimate the cost-effectiveness of echinocandins particularly for the aforementioned high-risk patients.

Furthermore, the cost estimates that only included the costs incurred during hospitalization may be underestimated (e.g., lack of long-term economic consequences of treatment or disease). However, because the final results of our interest were presented in the incremental costs between two treatment groups (i.e., a difference in cost estimates between two groups), the exact long-term economic consequences of treatment or disease might offset in the comparison between groups.

Second, our model estimates based on clinical trials might be different from what occurs in practice. Future studies that incorporate actual use of medical resources, including antifungal consumption, additional intervention for treatment failure or drug-associated adverse reactions, and treatments effective against drug-resistant microbes, should provide more valuable information and better reflect actual practice.

Third, although expert opinions are often used when there are no other sources of data available (i.e., LOS [23, 24]) and are commonly seen in pharmacoeconomic studies [9–11], this approach might bias the study results. Thus, we conducted sensitivity analyses and found that the cost-effectiveness results were robust to different values of LOS value.

Fourth, with regarding to the cost-effectiveness analyses specific to individual *spp.*(i.e., *C. albicans* and non-*albicans Candida spp.*), the efficacy data (i.e., success and mortality rates) of “non-echinocandins” (Table 1) were obtained from Andes et al.’s study [7] in which non-echinocandins included polyenes (i.e., amphotericin B and liposomal amphotericin B), and triazoles (i.e., fluconazole and voriconazole). In contrast, the efficacy data for echinocandins were primarily based on Reboli et al.’s study [8] which only assessed the efficacy of anidulafungin for *C. albicans* and non-*albicans Candida spp.*, respectively. This was done because very limited published studies reported the efficacy of echinocandins for individual *spp.*. However, since the efficacies of individual echinocandins appear to be similar [22], the data from anidulafungin might be representative of echinocandins.

Moreover, all efficacy data (i.e., treatment success) and model assumptions (i.e., average weight of patients receiving liposomal amphotericin B) were from other countries, which might not be applicable to an Asian population (e.g., Taiwan). We found data from Asian countries but the data varied by country (Additional file 1: Tables S4 and S5) and were different from those in international studies (e.g., Mills et al. [22], Reboli et al. [8]). Hence, an effectiveness study of antifungal treatments in an Asian population is needed to enable future cost-effectiveness research specific to Asia. The parameters of treatment efficacy (i.e., treatment success, morality rate) might be different depending on the length of the evaluation period. The cost-effectiveness model applied here used a 5-day period to define treatment success and a 6-week period to measure mortality associated with treatment. However, these efficacy data (i.e., survival) might be different if a longer evaluation period is chosen. Also, the treatment success and mortality data in the present study were based on a meta-analysis study [22], which pooled data based on different evaluation periods. Hence, detailed efficacy data associated with a specific evaluation period is needed. Finally, this economic evaluation was conducted from the perspective of a medical payer, and thus only direct medical costs were included. Further study that considers all economic consequences of disease and treatment (e.g., indirect costs such as productivity losses) is anticipated to give a broader view from a societal perspective.

## Conclusion

In summary, echinocandins are the dominant pharmacoeconomic alternative to fluconazole from Taiwan’s healthcare system perspective for treating invasive candidiasis. The clinical efficacy of antifungal therapy (i.e., mortality and treatment success rate) is the most influential determinant for the results of cost-effectiveness analysis. In the case of echinocandins, anidulafungin is appears to be the dominant option because of its higher efficacy at a lower total cost in the treatment of invasive candidiasis.

## Additional file

Additional file 1: Figure S1. Incremental cost-effectiveness ratio (ICER) under assumption that length of IV treatment for success and survival is 30 days. **Figure S2.** Incremental cost-effectiveness ratio (ICER) for comparison between echinocandins (anidulafungin as reference). **Figure S3.** Incremental cost-effectiveness ratio (ICER) based on efficacy data from Reboli et al.’s study [8]. **Table S4.** Summary of basic information on selected articles in Asia. **Table S5.** Summary of the results from systemic review and comparison to global data [25–31]. (DOCX 327 kb)

## Abbreviations

d-AmB: Amphotericin B deoxycholate
GDP: Gross domestic product
IC: Invasive candidiasis
ICER: Incremental cost-effectiveness ratio
IV: Intravenous
LOS: Length of hospital stay
LY: Life-years
