# Supplementary material for: Pharmacoeconomic analysis of antifungal therapy for primary treatment of invasive candidiasis caused by Candida albicans and non-albicans Candida species
Source: BMC Infect Dis. 2017 Jul 10;17:481. doi: 10.1186/s12879-017-2573-8 (PMC5504557; doi:10.1186/s12879-017-2573-8)
Supplement: Additional file 1: Figure S1. — Incremental cost-effectiveness ratio (ICER) under assumption that length of IV treatment for success and survival is 30 days. Figure S2. Incremental cost-effectiveness ratio (ICER) for comparison between echinocandins (anidulafungin as reference). Figure S3. Incremental cost-effectiveness ratio (ICER) based on efficacy data from Reboli et al.’s study [8]. Table S4. Summary of basic information on selected articles in Asia. Table S5. Summary of the results from systemic review and comparison to global data [25–31]. (DOCX 327 kb) [file 12879_2017_2573_MOESM1_ESM.docx]

**Additional file 1: Table S1. Incremental cost-effectiveness ratio (ICER) under assumption that length of IV treatment for success and survival is 30 days**

| **Invasive candidiasis** | **First-line treatment** | **Total  cost** | **Incremental cost** | **Total  life-years** | **Incremental  life-years** | **ICER** |
| --- | --- | --- | --- | --- | --- | --- |
| **All *Candida* species** | Fluconazole (ref.) | 5,499 | - | 6.52 | - | - |
|  | Anidulafungin | 11,118 | 5,619 | 7.23 | 0.70 | 8,015 |
|  | Micafungin | 11,857 | 6,358 | 5.55 | -0.98 | -6,506 |
|  | Caspofungin | 15,755 | 10,256 | 6.03 | -0.49 | -20,871 |
| ***Candida albicans*** | Non-echinocandins (ref.) | 25,188 | - | 5.97 | - | - |
|  | Echinocandins | 11,916 | -13,273 | 7.39 | 1.42 | -9,365 |
| **Non-*albicans Candida* spp.** | Non-echinocandins (ref.) | 11,487 | - | 4.56 | - | - |
|  | Echinocandins | 14,370 | 2,882 | 6.75 | 2.19 | 1,317 |

Notes: cost value is presented in 2015 US dollars.
 Liposomal amphotericin B used as alternative for those who failed first-line treatment.

**Additional file 1: Table S2. Incremental cost-effectiveness ratio (ICER) for comparison between echinocandins (anidulafungin as reference)**

| **Invasive candidiasis** | **First-line treatment** | **Total  cost** | **Incremental cost** | **Total  life-years** | **Incremental  life-years** | **ICER** |
| --- | --- | --- | --- | --- | --- | --- |
| **Assuming that length of IV treatment for success and survival is 14 days** | | | | | | |
| **All *Candida* species** | Anidulafungin (ref.) | 6,799 | **-** | 7.23 | **-** | **-** |
|  | Micafungin | 7,171 | 372 | 5.55 | -1.68 | -221 |
|  | Caspofungin | 9,211 | 2,412 | 6.03 | -1.20 | -2,010 |
| **Assuming that length of IV treatment for success and survival is 30 days** | | | | | | |
| **All *Candida* species** | Anidulafungin (ref.) | 11,118 | **-** | 7.23 | **-** | **-** |
|  | Micafungin | 11,857 | 739 | 5.55 | -1.68 | -440 |
|  | Caspofungin | 15,755 | 4,637 | 6.03 | -1.20 | -3,864 |

Notes: cost value is presented in 2015 US dollars. Liposomal amphotericin B used as alternative for those who failed first-line treatment.

**Additional file 1: Table S3. Incremental cost-effectiveness ratio (ICER) based on efficacy data from Reboli et al.’s study^8^**

| **Candidemia/  invasive candidiasis** | **Total  cost** | **Incremental cost** | **Total  life-years** | **Incremental  life-years** | **ICER** |
| --- | --- | --- | --- | --- | --- |
| Fluconazole (ref.)* | 4,825 |  | 6.26 |  |  |
| Anidulafungin* | 9,772 | 4,947 | 7.04 | 0.78 | 6,310.01 |
| Fluconazole (ref.) † | 4,315 |  | 6.26 |  |  |
| Anidulafungin† | 7,053 | 2,738 | 7.04 | 0.78 | 3,492.01 |

* Model inputs were from Reboli et al.’s study^8^ (after 6-week follow-up), in which success rate of anidulafungin is 55.9%, success rate of fluconazole is 44.1%, mortality rate for anidulafungin is 22.8%, and mortality rate for fluconazole is 31.4%.

† Model inputs were from Reboli et al.’s study^8^ (end of intravenous therapy), in which success rate of anidulafungin is 75.6%, success rate of fluconazole is 60.2%, mortality rate for anidulafungin is 22.8%, and mortality rate for fluconazole is 31.4%.

Note: cost value is presented in 2015 US dollars.

**Additional file 1: Table S4. Summary of basic information on selected articles in Asia**

| **Reference (author, year, area/country)** | **Sample size (n)** | **Intervention (drug, switch, or add) (treatment duration)** | **Time to defined clinical success (days)** |
| --- | --- | --- | --- |
| Mootsikapun P., et al., 2013, Asia^25^ | 43 | Anidulafungin/ anidulafungin shift to voriconazole | 42 |
| Kazama I. and K. Furukawa, 2003, Japan^26^ | 59 | Fluconazole/amphotericin B/fluconazole shift to amphotericin B | N/A |
| Yang Z.T., et al., 2014, China^27^ | 121 | N/A | N/A |
| Lee S.C., et al., 2014, Taiwan^28^ | 70 | Fluconazole/ anidulafungin/ caspofungin/ micafungin single use or fluconazole shift to echinocandins for 1-4 weeks | 180 |
| Ruan S.Y., et al., 2008, Taiwan^29^ | 40 | Fluconazole single use or shift to amphotericin B/ caspofungin or caspofungin shift to amphotericin B | N/A |
| Chen LY, et al., 2011, Taiwan^16^ | 871 | N/A | N/A |
| Chen PY, et al., 2014, Taiwan^30^ | 504 (comparison 2002, 2010 health-care infection) | Amphotericin B/ lipid form amphotericin B/ fluconazole/ voriconazole/ echinocandins/ combination | N/A |
| Wu S.P., et al., 2002, Taiwan^31^ | 109 (nosocomial infection) | Fluconazole/ amphotericin B/ combination | N/A |
| Ko L.L. and S.C. Chang, 2010, Taiwan^17^ | 126 | Triazoles: fluconazole, voriconazole; polyenes: amphotericin B, liposome form; echinocandins: caspofungin, micafungin | N/A |

**Notes:**

- Type of study design: only Mootsikapun P., et al.^25^ is prospective trial, others are retrospective design
- Only two studies (Mootsikapun P., et al.^25^ and Lee S.C., et al., 2014, Taiwan^28^) examined clinical success of treatment, where the criteria of clinical success had to meet both clinical and microbiological success.
- Mortality due to candidemia: Lee S.C., et al., 2014, Taiwan^28^, Ruan S.Y., et al., 2008, Taiwan^29^, Chen PY, et al., 2014, Taiwan^30^, and Wu S.P., et al., 2002, Taiwan^31^; all-cause mortality: Ruan S.Y., et al., 2008, Taiwan^29^
- Healthcare-associated infection: Chen PY, et al., 2014, Taiwan^30^; Nosocomial infection: Wu S.P., et al., 2002, Taiwan^31^ Healthcare-associated infection refers to CDC definition: “healthcare-associated infections (HAIs) include central line-associated bloodstream infections, catheter-associated urinary tract infections, and ventilator-associated pneumonia. Infections may also occur at surgery sites, known as surgical site infections.”
- Study in ICU patients: Ruan S.Y., et al., 2008, Taiwan^29^

**Additional file 1: Table S5. Summary of the results from systemic review and comparison to global data**

| **Reference (author, year, area/country)** | **Clinical success** | **Mortality** | **Averse drug reaction** | **LOS** |
| --- | --- | --- | --- | --- |
| **Asian studies** |  |  |  |  |
| Mootsikapun P., et al., 2013, Asia^25^ | Anidulafungin  MITT:* 73.8%  PP:* 79.4% | All-cause mortality : 33% (n=14/43) | 23.3% (10/43) | Anidulafungin: 11 days and oral voriconazole: 12.6 days |
| Kazama I. and K. Furukawa, 2003, Japan^26^ | N/A | -day mortality (?)*  Fluconazole: 50% (n=17/34)  Ampohtericin B or Fluconazole shift to amphotericin B: 40% (n=8/20) | N/A | N/A |
| Yang Z.T., et al., 2014, China^27^ | N/A | N/A | N/A | 35.3±168.4 days† |
| Lee S.C., et al., 2014, Taiwan^28^ | Anidulafungin 60.0% (n=18/30), caspofungin 88.9% (n=8/9), micafungin 71.4% (n=5/7), fluconazole 50% (n=9/18) | 30-day mortality:  Anidulafungin 26.7%, caspofungin 11.1%, micafungin 28.6%, fluconazole 38.9% | N/A | N/A |
| Ruan S.Y., et al., 2008, Taiwan^29^ | N/A | 30-day mortality:  Fluconazole 69% (n=11/16), fluconazole switch to amphotericin b 43% (n=9/21), fluconazole switch to caspofungin 100% (n=2/2), caspofungin switch to amphotericin B 100% (n=1/1) | N/A | N/A |
| Chen L.Y., et al., 2011, Taiwan^16^ | N/A | 30-day crude mortality 36.9% (n=321) | N/A | 38±101 days† |
| Chen P.Y., et al., 2014, Taiwan^30^ | N/A | 30-day mortality, 40.41% (n=127 in 2010) | N/A | N/A |
| Wu S.P., et al., 2002, Taiwan^31^ | N/A | 14-day mortality: 40.4% (n=44/ 109) | N/A | 5±94 days† |
| Ko, K.L. and S.C. Chang, 2010, Taiwan^17^ | N/A | 30-day mortality:57.1% (n=72/126), triazoles 52.2% (n=48/92), polyenes 63.6% (n=7/11), echinocandins 60% (n=9/15)‡ | N/A | 26.7±30.4 days† |
|  |  |  |  |  |
| Mills E.J., et al., 2009, global^22^ | Anidulafungin 77.49%, fluconazole 63%, amphotericin B 65.4%, caspofungin 76.1%, voriconazole 65.03%, micafungin 75.98%, liposomal amphotericin B 72.98% | All-cause mortality: anidulafungin 20.75%, fluconazole 28.44%, amphotericin B 30.93%, caspofungin 33.83%, voriconazole 25.8%, micafungin 39.16%, liposomal amphotericin B 39.99% | Nephrotoxicity: relative risk of echinocandins compared with amphotericin B: 0.31, relative risk of azoles compared with amphotericin B: 0.22 | N/A |

*Abbreviations: LOS: Length of Stay, MITT: Modified intend-to-treat group, PP: pre-protocol

†Calculated from original data, which is represented as follows: Yang Z.T., et al., 2014, China:^27^ length of hospitalization: 74.6±97.7 days, and time from admission to infection: 39.3±70.7 days; Chen L.Y., et al., 2011, Taiwan:^16^ length of hospital stay: 75±71 days, and pre-infection hospital stay: 37±30 days (surviving > 30 days: 35±28 days; dying within 30 days: 40±32 days); Wu S.P., et al., 2002, Taiwan:^31^ length of hospital stay: 82±128 days, and pre-infection hospital stay: 77.25±33.9 days; Ko, K.L. and S.C. Chang, 2010, Taiwan:^17^ length of hospital stay: 64.1±118.2 days, and pre-infection hospital stay: 37.3±116.4 days.

‡Patients who discharged based on their own opinion and then within 30 days progressed to a critical state are counted as mortality.

**Additional file 1: Figure S1. echinocandins vs. non-echinocandins for C. *albicans* (or non-*albicans*) infection**


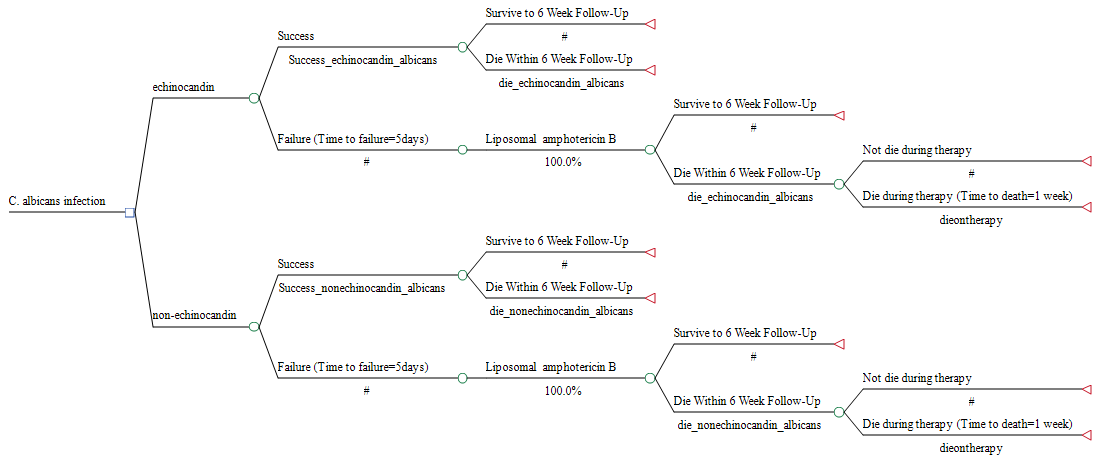


Abbreviations: success_echinocandin_albicans: success rate for echinocandins for *C. albicans* infection, success_nonechinocandin_albicans: success rate for non-echinocandins for *C. albicans* infection, die_echinocandin_albicans: mortality rate for echinocandins for *C. albicans*, die_nonechinocandin_albicans: mortality rate for non-echinocandins for *C. albicans*, dieontherapy: the percentage of patients who died during therapy (they had not completed the treatment before death; died during treatment

**Additional file 1: Figure S2. Tornado diagram for 1-way sensitivity analysis of echinocandins vs. non-echinocandins for *C. albicans* and non*-albicans* infection (x axis presents values of incremental cost-effectiveness ratio; ICER, cost per life-year gained. Cost value is presented in 2015 US dollar.)**


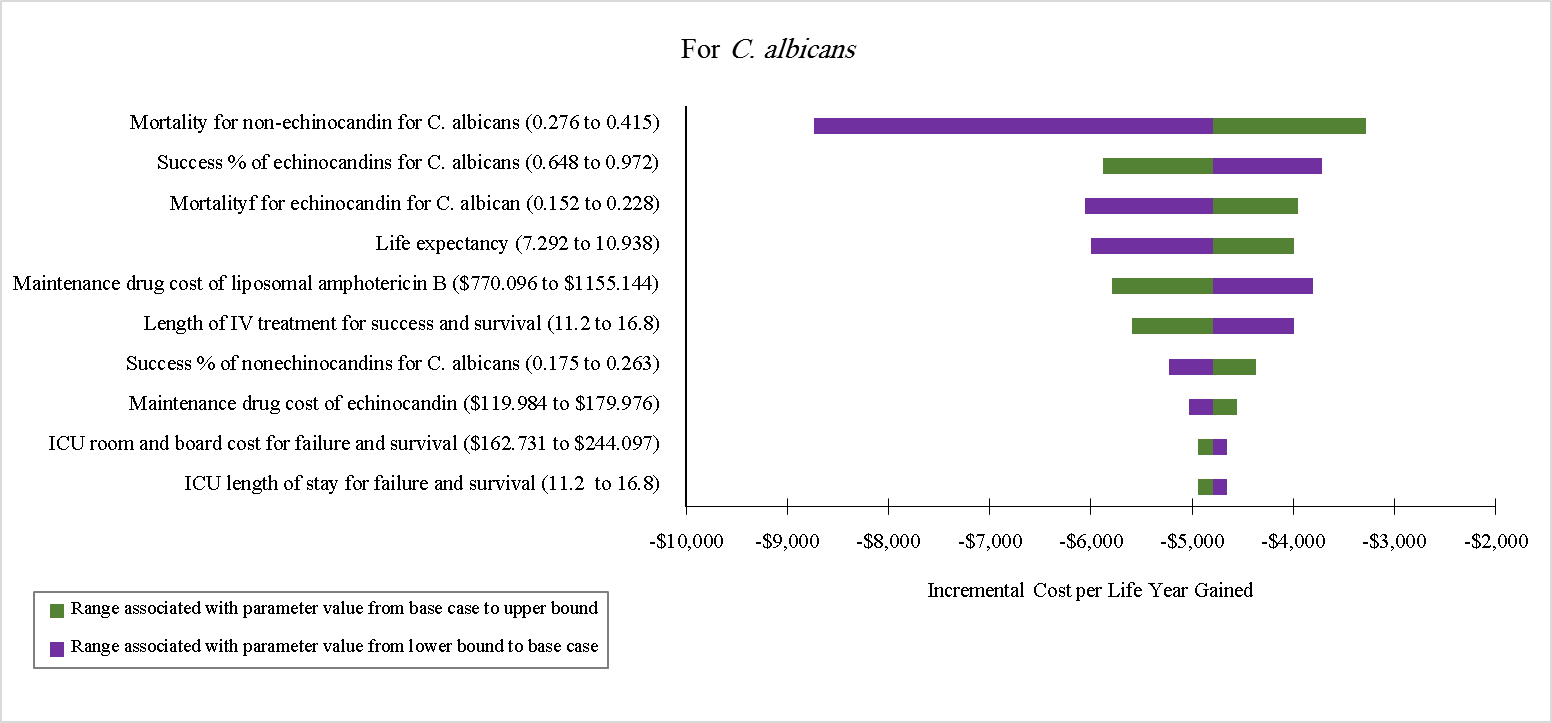

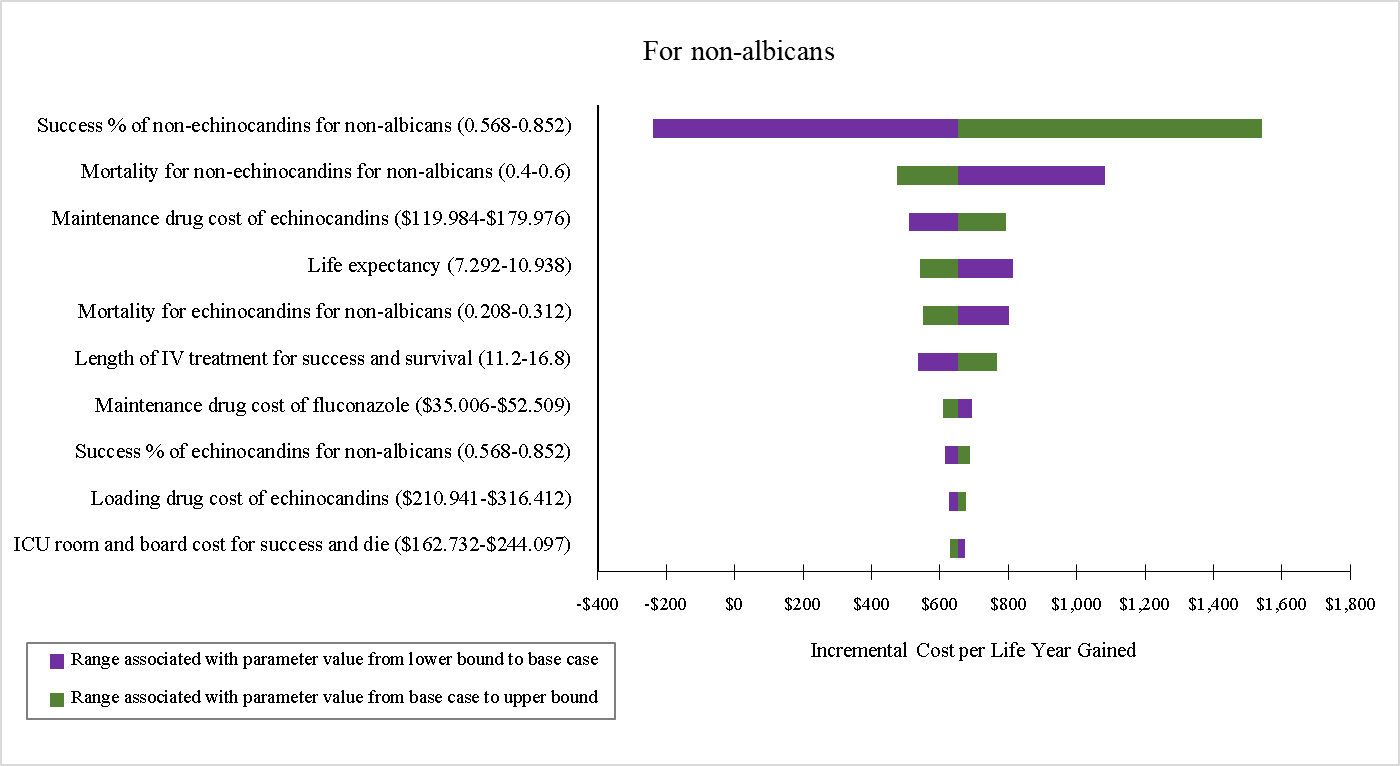


Base case: -$4,796

Base case: $652

0.606

|  |  |
| --- | --- |

**Additional file 1: Figure S3. One-way sensitivity analysis of success rate of non-echinocandins for *C*. *albicans* (echinocandins vs. non-echinocandins)**


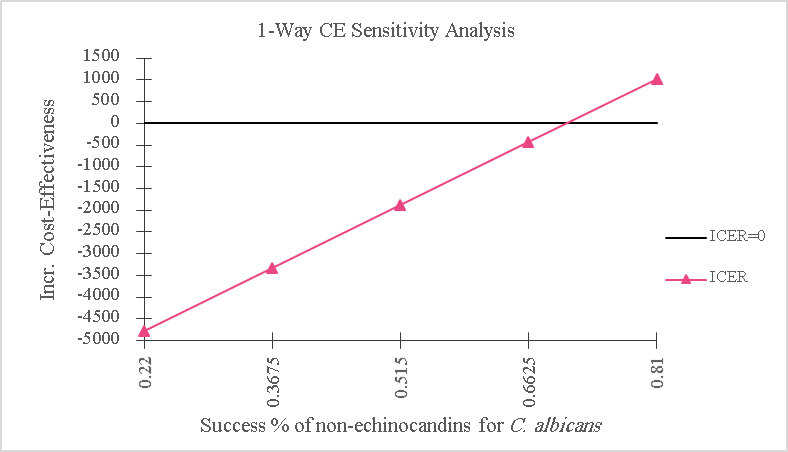


0.706

-$4,795.85
